# Molecular Epidemiology, Genetic Diversity, and Antifungal Susceptibility of Major Pathogenic Dermatophytes Isolated From Human Dermatophytosis

**DOI:** 10.3389/fmicb.2021.643509

**Published:** 2021-06-04

**Authors:** Zahra Salehi, Masoomeh Shams-Ghahfarokhi, Mehdi Razzaghi-Abyaneh

**Affiliations:** ^1^Department of Mycology, Faculty of Medical Sciences, Tarbiat Modares University, Tehran, Iran; ^2^Department of Mycology, Pasteur Institute of Iran, Tehran, Iran

**Keywords:** dermatophytes, dermatophytosis, molecular epidemiology, antifungal susceptibility, genetic diversity, multilocus sequence typing

## Abstract

**Background:**

Dermatophytes are a homogeneous group of species with low genetic diversity, and there are still many uncertainties about the boundaries among species.

**Objectives:**

Aiming at clarifying the relationships among species in the genus and introducing suitable genes for multilocus sequence typing (MLST), a new MLST scheme approach was developed to characterize the major pathogenic dermatophytes.

**Methods:**

We performed maximum parsimony (MP), MrBayes, RAxML, and eBURST analyses, based on the MLST scheme to scrutinize the evolution within 95 clinical isolates and four reference strains belonging to the four major dermatophytes species. Then, the discriminatory power, pairwise genetic distances, ratio dN/dS, and sequence types (STs) of these isolates were determined. Also, to study taxonomy, sequences of the internal transcribed spacer (ITS), *Beta-tubulin* (*BT2***),** and *translation elongation factor 1*-α (*TEF-1*α) genes of other dermatophytes species available in the GenBank were analyzed.

**Results:**

Findings of the present study indicated that three genes: *BT2*, ITS, and *TEF−1*α, which showed the greatest diversity among dermatophyte species, were suitable for MLST. The most prevalent STs were seen among the species of *Trichophyton interdigitale*. Also, two new genotypes, i.e., XXVII and XXVIII, were introduced for *T. interdigitale* and *Trichophyton mentagrophytes*. The least informative sites were found in *Epidermophyton floccosum*, *Trichophyton rubrum*, and *T. mentagrophytes*, while the most informative sites were observed in *T. interdigitale*. Furthermore, the most informative locus was *TEF-1*α. The phylogenetic tree, constructed by the combination of the three genes, shows a new topological pattern that confirms the derivation of the anthropophilic and zoophilic genera from the geophilic genus. Also, the phylogenetic analyses and pairwise distances of the combination of the three loci showed that *Trichophyton tonsurans* and *Trichophyton equinum* were a species complex, where *T. equinum* is derived from *T. tonsurans*.

**Conclusions:**

Results of this study showed that MLST is very effective in determining the boundaries between species and taxonomy. Considering that there is no database for MLST dermatophytes, further studies are needed to determine the suitable genes for MLST. Also, the determination of STs in epidemiological studies and raising epidemiological information are helpful. This study was a new starting point to determine the ST and a foundation for a dermatophyte MLST database.

## Introduction

Dermatophytes are a group of related keratinophilic fungi categorized by several genera, namely, *Trichophyton*, *Microsporum*, *Epidermophyton*, *Nannizzia*, *Paraphyton*, *Lophophyton*, and *Arthroderma* based on the new taxonomy (de Hoog et al., 2017). They play a significant role as pathogens in humans and animals (Summerbell et al., 1999; Cafarchia et al., 2013; Zarrin et al., 2015; Mochizuki et al., 2016). Concerning the recent epidemiologic surveys in Tehran, dermatophytosis was found to be 19.7%, and the major species involved the *Trichophyton mentagrophytes/Trichophyton interdigitale* species complex, *Trichophyton rubrum*, *Trichophyton tonsurans*, and *Epidermophyton floccosum* (Sadeghi et al., 2011; Zamani et al., 2016). It is believed that the PCR-based assays are quick and useful tools for the diagnosis and differentiation of isolates at the species and subspecies level, especially for the atypical isolates (Kac, 2000; Graser et al., 2008). Gene sequencing is one of the most informative and well-known techniques for fungal diagnosis (Susca et al., 2013). Multilocus sequence typing (MLST) is a very stable and reproducible technique that determines the isolates of the microbial species using the DNA sequences of multiple housekeeping genes (Bougnoux et al., 2002; Bain et al., 2007; Vanhee et al., 2010; Bernhardt et al., 2013). MLST results are unequivocal, and sequence data can be shared and compared between different laboratories (Bernhardt et al., 2013). More specifically, the multilocus sequence can distinguish between the individual strains and other isolates, and evaluate the relationships, recombination, and mutation rates among microorganisms, which are classified under the same genus (Bougnoux et al., 2002). The phylogenetic species concepts and phylogenetic analysis based on the internal transcribed spacer (ITS) regions contribute to the improvement of the taxonomy. At present, the sequence database of the ITS region is considered to be the gold standard for dermatophytes (Graser et al., 2008; Anzawa et al., 2011). Sequencing of the *Beta-tubulin* (*BT2*), *translation elongation factor 1-*α (*TEF-1*α) genes, *calmodulin* (*CaM*), or sequencing the D1/D2 domain of the large-subunit rRNA gene, as well as the ITS region, can reliably identify the species (Li et al., 2008; Anzawa et al., 2011; Mirhendi et al., 2014, 2016; Rezaei-Matehkolaei et al., 2014; Ahmadi et al., 2016). The results of previous studies emphasize that very few genetic changes happened in the evolutionary history of dermatophytes (Makimura et al., 1999; de Hoog et al., 2017; Persinoti et al., 2018; Zhan et al., 2018). Therefore, to attain more accurate results, exploring the SNP analysis of these genes is suggested. Fungal taxonomy development is a controversial issue, and the information in this field is not sufficient for the dermatophyte species. The dermatophyte species has been recently revised based on the MLST (de Hoog et al., 2017). For example, the recent study showed that *T. mentagrophytes* and *T. interdigitale* belong to the same phylogenetic species (Pchelin et al., 2016). Yet, there remain many uncertainties concerning the boundaries between dermatophytes species. Data obtained from MLST help to resolve species boundaries (O’Donnell et al., 2004). Therefore, more detailed studies are necessary for determination purposes. These led to the following questions: (i) Are *T. tonsurans* and *Trichophyton equinum* distinct species? and (ii) Are *T. rubrum* and *Trichophyton violaceum* belong to a complex?

Besides, to the best of the researchers’ knowledge, there are a few studies with the MLST of dermatophytes (Probst et al., 2002; de Hoog et al., 2017; Pchelin et al., 2016; Persinoti et al., 2018; Zhan et al., 2018). In recent years, the trend toward this technique has been increased to investigate dermatophyte species. Moreover, previous studies on MLST have not addressed the genotypes, sequence types (STs) species, discriminatory power, ratio dN/dS, and informative sites (Probst et al., 2002; Pchelin et al., 2016; de Hoog et al., 2017). The present study focuses on the determination of the ST dermatophyte species, and is specially designed for (i) providing a better understanding of the species; (ii) highlighting the molecular diversity and genotypes characterized by major dermatophyte species on seven loci: *BT2*, *TEF-1*α, *ACT*, *CaM*, *HSP70*, D1/D2, and ITS; (iii) evaluating the genes used for the MLST approach (iv) specifying the ST dermatophyte species; and (v) investigating the association between genotypes and STs with an antifungal sensitivity profile (vi) offering a closer look at the taxonomy of dermatophytes based on the combination of the three genes: ITS, *BT2*, and *TEF-1*α.

## Materials and Methods

### Fungal Isolates

Clinical samples were selected from the Pathogenic Fungi Culture Collection of Pasteur Institute, Iran. Initially, the phenotypically distinct isolates were identified, followed by re-identification through the use of the sequencing related to the ITS region of rDNA. To this end, a total of 99 strains consisting of 95 dermatophyte clinical isolates, including *T. rubrum* (*n* = 19), *T. interdigitale* (*n* = 20), *T. mentagrophytes* (*n* = 6), *T. tonsurans* (*n* = 28), *E. floccosum* (*n* = 22), as well as standard strains provided from the Persian Type Culture Collection (PTCC) of *T. Rubrum* PTCC 5143, *T. mentagrophytes* PTCC 5054, *T. tonsurans* CBS 130924, and *E. floccosum* CBS 767.73 were used in the sequence analysis. Clinical features of these isolates are provided in Supplementary Table 1. All the strains were isolated from the skin and hair specimens. The names of the species were determined according to de Hoog et al. (2017).

### DNA Extraction and Gene Amplification

All fungal strains were cultured on a mycobiotic agar (Merck, Germany) and incubated at 28°C for 4–7 days. DNA was extracted using the phenol-chloroform-isoamyl alcohol according to Makimura et al. (1999).

PCRs were carried out in 50 μl reaction volumes for *TEF-1*α, *BT2, CaM*, *ACT*, *HSP70*, ITS, and the D1/D2 fragments. Each mixture contained 25 μl of Premix (Ampliqon, Denmark), 3 μl of DNA template, and 0.8 μM of each primer, and the remaining volume was filled with water to reach a final volume of 50 μl. Negative controls (water instead of the fungal DNA) were added to each PCR. PCR amplification with different primer pairs was performed for all the isolates, following the seven loci adapted from the earlier genotyping studies (Table 1). The reaction mixture was initially denatured at 95°C for 5 min, followed by 30 cycles of 30 s at 94°C, annealing at changes in temperatures (from 55 to 67°C) for 40 s, 45 s at 72°C, and a terminal extension step of 72°C for 5 min. Five microliters of the PCR products was electrophoresed on 1% agarose gel in TAE buffer (Tris 40 mM, acetic acid 20 mM, and EDTA 1 mM), and photographed under ultraviolet irradiation.

**TABLE 1:** Oligonucleotide primers used for the molecular identification of dermatophytes.

**Locus**	**Primers**	**Sequence (5′–3′)**	**Annealing temperature (°C)**	**References**
*TEF-1*α	Forward	CACATTAACTTGGTC GTTATCG	58	Mirhendi et al., 2014
	Reverse	CATCCTTGGAGAT ACCAGC		
*BT2*	Forward	AACATGCGTGAGA TTGTAAGT	56	Rezaei-Matehkolaei et al., 2014
	Reverse	ACCCTCAGTGTAGT GACCCTTGGC		
*CaM*	Forward	TGTCCGAGTACA AGGAAGC	60	Ahmadi et al., 2016
	Reverse	TTACAATCAAT TCTGCCGTC		
*ACT*	Forward	TCTTCGAGACC TTCAACGCC	67	Probst et al., 2002
	Reverse	AAGCCACCGATC CAGACG		
*HSP70*	Forward	GTGGCTTCCCA GGTGCTG	55	Probst et al., 2002
	Reverse	AATGATTTCAGTAAC CGACCC		
ITS	Forward	TCCGTAGGTGAACC TGCGG	58	White et al., 1990
	Reverse	TCCTCCGCTTATTG ATATGC		
D1/D2	Forward	GCATATCAATAAGCGGA GGAAAAG	60	Kurtzman and Robnett, 1997
	Reverse	GGTCCGTGTTTCA AGACGG		

### Sequencing

All seven genes were successfully amplified and sequenced in forward and reverse for all 99 isolates. The PCR products were subjected to the ABI PRISM BigDye Terminator Cycle Sequencing Ready Reaction Kit (Applied Biosystems, Foster City, CA, United States). The forward and reverse sequences of each sample were subjected to ClustalW pairwise alignment using the MEGA7.0.21 software, and edited manually to improve the alignment accuracy (Kumar et al., 2001). Two-web databases of the CBS (for sequencing ITS regions) and BLASTn (for six other gene fragments) were used for identifying and comparing the fungi. All isolates were verified to the species level by sequencing the ITS regions of rDNA.

### Phylogenetic Analysis

The bioinformatics data were analyzed and used to diagnose the interspecies and intraspecies nucleotide variation of seven loci in all of the 99 isolates in this study. The sequence data from each gene were aligned using the MEGA7.0.21 software; then, they were manually adjusted and concatenated (4,620 bp). Also, the sequences were analyzed by maximum parsimony (MP) for finding the informative sites of genes of the isolates that belonged to each species and PAUP version 4.0b109 to the Bayesian analysis for drawing the phylogenetic tree (Swofford, 2002). The best-fit model of molecular evolution was estimated in the jModelTest 2.1.10 (Darriba et al., 2012). The programs MrBayes version 3.2 (Ronquist et al., 2012) and RAxML version 8.2 (Miller et al., 2010; Stamatakis, 2014) were run on the CIPRES Science Gateway. Subsequently, two simultaneous analyses with eight Metropolis-coupled Markov chain Monte Carlo (MCMC) chains and incremental heating of 0.2 were run for 20 million generations which were sampled in every 1,000 generations. The convergence of parameter estimates was verified and effective sample sizes were obtained >200 for all parameters using the Tracer version 1.6 (Drummond and Rambaut, 2007). Maximum-likelihood (ML) analyses were performed using the RAxML. Optimization in RAxML was carried out using the GTRCAT option. Bootstrap values for maximum likelihood were 1,000 replicates with one search replicate per bootstrap replicate. This analysis was performed for 99 isolates, an individual gene, and a combination of seven genes. *Fusarium solani* was used as an outgroup to root the dendrogram.

### Phylogenetic Reconstruction

First, species involving all three sequences, *BT2*, *TEF-1*α, and ITS, were deposited in the GenBank and identified for phylogenetic reconstruction. Then, these sequences became the datasets. The combined multilocus (*BT2*, *TEF-1*α, and ITS) dataset of the 99 isolates of this study along with the published sequences dataset was aligned using the MEGA7.0.21 software. ML analyses of the mentioned sequences were performed using the RAxML as described above. *F. solani* was used as an outgroup in a phylogenetic tree, constructed with Maximum likelihood using the RAxML v. 8.0.0 under the GTRCAT model and 1,000 bootstrap replications. Bootstrap support above 95% is shown above the branches.

### Genotyping

Since ITS-genotyping is only determined for the *T. interdigitale*/*T. mentagrophytes* species complex and not for other species and genes, we used the nomenclature from the studies by Heidemann et al. (2010) and Taghipour et al. (2019) for the ITS-genotyping of the *T. interdigitale*/*T. mentagrophytes* complex. We chose the borders of the ITS region of *T. interdigitale* as deposited under the accession number MK312735 for homogeneity, and did not consider the four nucleotides (GGTT) at the 5′−end of our deposited sequences in the GenBank. For other species and genes in this study, the numeration was used, starting from 1.

### MLST Analyses

Because an MLST database for dermatophytes does not exist, the ST numbers started from 1. The ST was put in numerical tags as a contract, which was based on the case suggested by Bougnoux et al. (2002).

According to the MLST standards, particular allele sequences for each of the housekeeping genes were labeled as a genotype, and each different combination of genotypes was determined as a distinct ST. STs were also analyzed using the eBURST package^1^ to determine the possible relationships between isolates based on the single allele differences (Feil et al., 2004; Spratt et al., 2004). The discriminatory power was calculated according to Hunter’s formula (Hunter, 1990). Pairwise genetic distances (p-distances) between species were calculated using the MEGA7.0.21. The possibility of selective pressure at each of the loci was computed by the ratio of non-synonymous nucleotide substitutions (dN/dS) calculated by Nei and Gojobori (1986). Fisher’s exact test was used to determine the associations between the ST and anatomical origin, age, gender, and genotype using the statistical SPSS package (version 19). Values with *P* < 0.05 were considered significant.

### Antifungal Susceptibility Testing

Susceptibility of 95 clinical isolates and four reference strains to griseofulvin, lanoconazole, butenafine (Sigma-Aldrich), and terbinafine (Dr. Reddy’s Laboratories) were determined according to the CLSI M38-A2 broth microdilution method (Wayne, 2008). MIC results were visually read after 4 days of incubation at 35°C. The MIC was defined as the point at which the growth of dermatophyte was inhibited by 80% or more reduction for eight antifungals after a visual inspection in comparison with the control. MIC range, geometric mean, MIC_50_, and MIC_90_ were then calculated for the isolates that belonged to each species. All tests were performed in duplicate. *Candida krusei* (ATCC 6258) and *Candida parapsilosis* (ATCC 22019) were used as quality controls.

## Results

### Fungal Isolates

*Trichophyton interdigitale* and *T. mentagrophytes* were often isolated from tinea pedis, and *E. floccosum* was the most common cause of tinea cruris. *T. rubrum* and *T. tonsurans* are distributed among different areas (Supplementary Table 1).

### Sequencing and Gene Diversity

**FIGURE 1:**
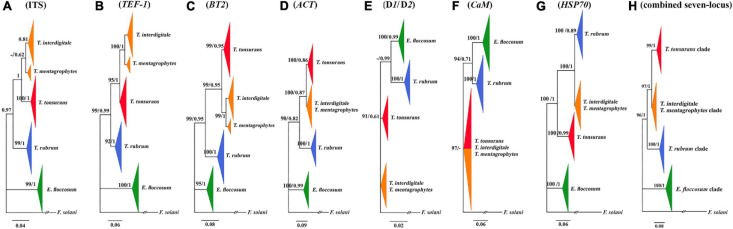
Comparison of seven trees constructed by the Bayesian analysis. Phylogenetic analysis of ITS **(A)**, *TEF-1*α **(B)**, *BT2* **(C)**, *ACT* **(D)**, D1/D2 **(E)**, *CaM* **(F)**, *HSP70* **(G)**, and the consensus trees constructed by the Bayesian analysis from the combined seven-locus **(H)** of the 95 clinical isolates, four reference strains, and use of *F. solani* as the outgroup. The bootstrap percentages of the ML analysis and posterior probabilities values are presented at the node (PP/BS). Values less than 70% bootstrap support in the ML analysis and less than 0.5 posterior possibility in the Bayesian analysis were indicated with a hyphen.

PCR of the ITS, D1/D2, *BT2*, *TEF-1*α, *HSP70, ACT*, and *CaM* genes of the 99 isolates and the standard strains showed bands, sizes of which ranged from 350 to 800 bp. The nucleotide substitutions of different species by the gene regions are represented in Table 2. The gene regions without a nucleotide change are not included in Table 2. The *HSP70* sequences did not show any intraspecies variation in all species (Figure 1). All the newly generated sequences were deposited in the GenBank (Supplementary Table 2).

**TABLE 2:** Characteristics and the nucleotide substitutions of the housekeeping loci studied.

**Species**	**Locus**	**Amplicon size (bp)**	**Strains**	**Nucleotide substitutions**	**Genotypes**	**SNPs (No.)**	**nSNP (No.)**
*T. interdigitale*	ITS	627*	-TI 7, TI 13	-C158T (XXVII)**	2	1	NCP
	*TEF-1*α	768	-TI 15, TI 17	-C566T	5	4	3
			-TI 18	-C704G			
			-TI 19	-C705G			
			-TI 20	-T709G			
	*BT2*	790	-TI 16	-C72T	3	3	1
			-TI 13	-G129C, G135C			
	*ACT*	687	-TI 14	-C141T	2	1	-
	D1/D2	617	-TI 7, TI 13	-C96T, C449T	3	3	NCP
			-TI 1, TI2	-T441C			
			-TI4, TI 5, TI6, TI 7	-T441C			
			-TI 13, TI16	-T441C			
	*CaM*	664	-TI 15, TI 21	-G414C, G587A	2	2	2
*T. mentagrophytes*	ITS	627*	-TM 3	-C158T (XXVIII)**	2	1	NCP
	*TEF-1*α	768	-TM 4	-C704G	2	1	1
	D1/D2	617	-TM 2, TM 3, TM 4	-T441C	2	1	NCP
*T. rubrum*	*TEF-1*α	737	-TR 18, TR 19, TR 20	-C340A,A503C	3	3	2
	*BT2*	794	-TR 17	-A514C, A583C	3	3	2
			-TR 18	-C270T			
	*ACT*	711	-TR 16	-G180T	2	1	1
*T. tonsurans*	*TEF-1*α	756	-TT 27	-T587C, T606C	4	3	2
			-TT 28	-T587C, C614T			
			-TT 29	-T606C, C614T			
*E. floccosum*	*TEF-1*α	732	-EF 3	-A249C	3	2	1
			-EF 22	-G511A			
	*BT2*	789	-EF 23	-C60G	2	1	-
	*ACT*	706	-EF 1, EF 2	-C679T	2	1	1

*The length of the fragment was 675 bp, which was reduced to 627 bp after alignment with the genotypes deposited in the GenBank (JX122216 and KP132819) and cropping at the beginning and end of the fragment.

**New genotypes.

*SNP, single-nucleotide polymorphisms; NCP, non-coding protein; nSNP, non-synonymous SNPs.*

**FIGURE 2:**
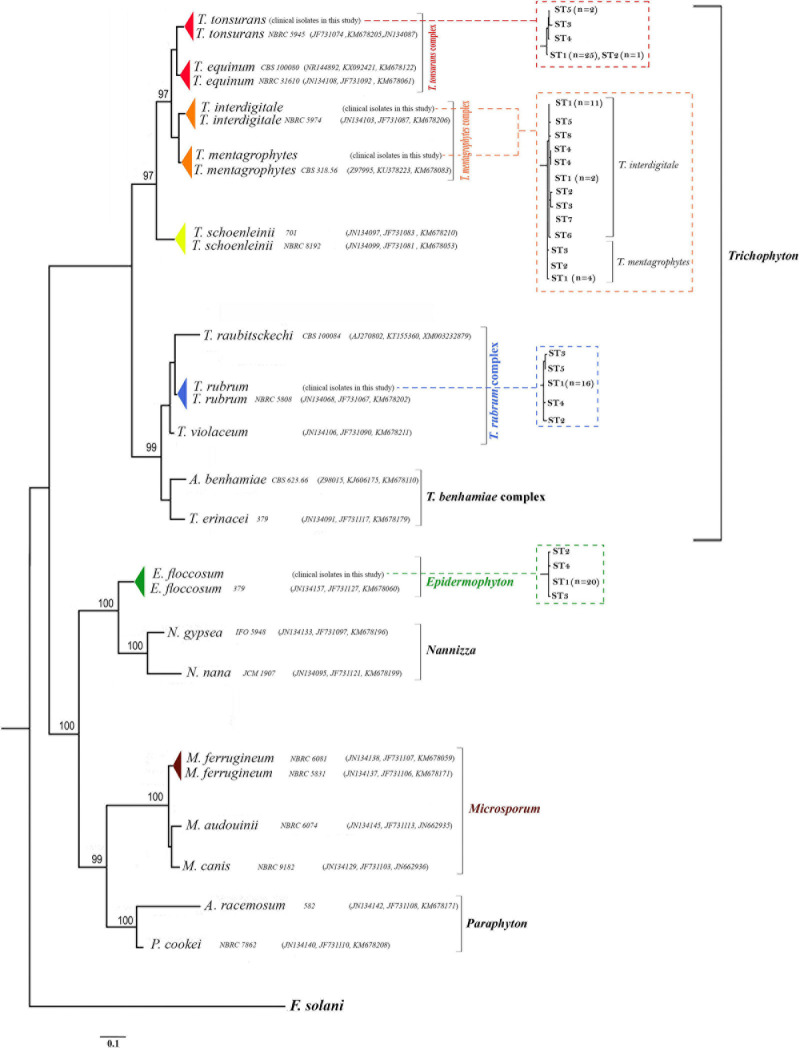
Phylogenetic relationships within dermatophytes. The consensus trees constructed by RAxML analysis from the combined three-locus ITS, *BT2*, and *TEF-1*α from clinical isolates and dermatophytes species available in the GenBank. The accession number profile was written in order ITS, *BT2*, *TEF-1*α loci. *F. solani* was used as an outgroup to root the dendrogram. The bootstrap value greater than 95% is shown above the branches. Also, the relationship between STs and taxa in RAxML analyses for the 99 dermatophyte species used in this study from the combined three-locus ITS, *BT2*, and *TEF-1*α is shown in the dot-box plots.

The characteristics of the multilocus obtained from the sequences of the PCR products from *T. interdigitale* loci were compared to those of the corresponding loci in *T. mentagrophytes*, *T. rubrum*, *T. tonsurans*, and *E. floccosum* (Table 2). The sequences were obtained from the combination of seven genes which contained 4,620 nucleotides. The *HSP70* region of *E. floccosum* was found to be the shortest gene with only 346 bp, while *BT2* was the longest gene of *T. rubrum* with 794 bp. The number of nucleotide substitutions from the dermatophytes genome sequence was studied, which were not conserved at the amino acid level, and the most amino acid changes were found in *T. interdigitale* (three) at the locus *TEF-1*α. For example, the *HSP70* sequences failed to reveal intraspecies variation in all species. Also, the ITS, *CaM*, and D1/D2 sequences did not show intraspecies variation in *T. rubrum*, adding that the ITS, *BT2*, *ACT*, *CaM*, and D1/D2 sequences did not show intraspecies variation in *T. tonsurans*. The species with no intraspecies variations (with only one genotype) are excluded from Table 2. In the combination of three loci (ITS, *BT2*, and *TEF*-*1*α), there was a relationship between STs and taxa (except for *T. tonsurans* that ST1 and 2 were in the same taxa and *T. interdigitale* that ST1 was distributed among the other STs) (Figure 2), whereas, in the combination of seven genes, this relationship was seen in 21 of the 27 singletons.

### Phylogenetic Analysis in Dermatophytes

A phylogenetic tree for a combined seven loci is shown in Figure 1. Based on the phylogenetic analysis, all isolated strains studies were placed in four supported clades (pp = 1). *E. floccosum* clade takes a basal position to the *Trichophyton* clades (Figure 1).

Individual *CaM* tree failed to separate the clade of *T. mentagrophytes/T. interdigitale* complex from the clade of *T. tonsurans* (Figure 1), while individual ITS, D1/D2, *BT2*, *TEF-1*α, *HSP70*, and *ACT* trees provided altogether the satisfactory resolution in four clades (Figure 1). The results of individual trees showed that the only individual ITS, *BT2*, and *TEF-1*α tree can distinguish between *T. mentagrophytes* and *T. interdigitale* in distinct clusters and *T. mentagrophytes* strains are placed in a basal position to the *T. interdigitale* (Figure 1).

In the data obtained from the MP analysis of seven loci, no informative site was observed to be *HSP70*, while the most informative locus was *TEF-1*α. Overall, these results showed that the most informative sites were found in *T. interdigitale* isolates (Table 3).

**TABLE 3:** The data obtained from the maximum parsimony analysis and phylogenetic analysis of seven loci.

**Species**	**Characters (bp)**	**PIC (No.)**	**AUT (No.)**	**ST (No.)**
*T. interdigitale*	4,608	7	8	12
*T. mentagrophytes*	4,612	1	3	5
*T. rubrum*	4,620	1	5	6
*T. tonsurans*	4476	3	-	6
*E. floccosum*	4,588	1	3	5

*PIC, parsimony-informative character; AUT, autapomorphy (a derived character unique to a particular taxon).*

### Phylogenetic Reconstruction

Deposited sequence of 22 strains in the GenBank was used for the phylogenetic reconstruction. Twenty-two standard strains were selected in a way that all three *BT2*, *TEF-1*α, and ITS sequences were deposited in the GenBank. These 22 sequences along with 99 isolates of this study were subjected to ClustalW pairwise alignment using the MEGA7.0.21 software (Figure 2).

Five genera introduced by the study of de Hoog et al. (2017) (*Epidermophyton*, *Microsporum*, *Nannizzia*, *Paraphyton*, and *Trichophyton*) were used in this classification, and other genera were not included in this classification due to the absence of the *TEF-1*α, *BT2*, and ITS sequences in the GenBank. The highest ratio of bootstrap-support (100%) was observed in groups *Epidermophyton*, *Microsporum*, *Nannizzia*, and *Paraphyton*, and the lowest belonged to *Trichophyton*.

Pairwise distances of the combination of the three loci (based on overall average) were examined, indicating a high degree of overlap (difference 0.001%) between the species *T. tonsurans* and *T. equinum* (results are not shown). Also, P-distances of the combination of the three genes showed homology of about 99.994% between *T. rubrum* and *T. violaceum* species. On the other hand, P-distances of the combination of the three genes showed significant differences between the species *N. nana* and *N. gypsea* with *E. floccosum* (results are not shown).

### Genotyping

A total of 19 out of 21 *T. interdigitale* isolates were classified in genotype II, and five out of six *T. mentagrophytes* isolates were classified in genotype II^∗^. A total of two new genotypes were identified in this study; one genotype was obtained in two *T. interdigitale* isolates (XXVII) and the other in one isolate of *T. mentagrophytes* (XXVIII).

Genotypes (except for the ITS-genotyping of *T. mentagrophytes/T. interdigitale* complex) and STs numerically start from 1 in the contract due to the absence of the MLST database for dermatophyte species. The positions of the single-nucleotide polymorphisms (SNPs), the different genotypes identified at each of the seven loci, and the determination of the genotype, as well as their frequency, are shown in Table 2. Besides, these numbers correlate with the genotype numbers listed in Supplementary Table 2 which represents how ST is determined and shows nucleotide diversity at the level of dermatophytes species. Regarding the genetic variability, the highest number of genotypes was found within the *T. interdigitale* (five genotypes) species (Supplementary Table 2 and Table 2). The phylogenetic analysis of species with only one genotype is excluded from Table 2. The analysis of 99 isolates yielded 34 STs which are illustrated in Supplementary Table 2. All in all, 27 STs were singletons (represented by a single isolate).

### MLST Analysis

The eBURST analysis was performed for five species. The discriminatory power of the MLST for *T. interdigitale*, *T. mentagrophytes, T. rubrum*, *T. tonsurans*, and *E. floccosum* was estimated to be 0.881, 0.9333, 0.4474, 0.3202, and 0.5217, respectively. The 32 SNPs among the seven loci led to 16 non-synonymous changes in encoding amino acids. Considering the species studied, dS occurred more frequently than dN; thus, the ratio of dN/dS was less than one for five loci (protein-coding): *E. floccosum*: −1.19, *T. interdigitale*: −1.18, *T. mentagrophytes*: −0.98 *T. rubrum*: −0.78, and *T. tonsurans*: −0.28. Pairwise distances of the combination of the seven loci (based on an overall average) to resolve the boundaries between the *Trichophyton* species are shown in Supplementary Table 3, whereas the sequence homology between the *T. interdigitale* and *T. mentagrophytes* species was noted to be greater than 99.999%. A comparison of these numbers indicates that these two species belong to a complex. The second-most similarities were observed between the species of *T. mentagrophytes/T. interdigitale* complex with *T. tonsurans*, and the most distant species were found to be *T. rubrum*.

By Fisher’s exact test, no significant association was found between ST and the anatomical source. Also, phylogenetic analysis of each locus did not show any statistically significant variation across clades, anatomical sources, and demographic information.

### Antifungal Susceptibility Testing

The results of antifungal susceptibility testing for the 99 isolates of dermatophytes against four antifungals are shown in Table 4. Overall, the lowest MIC were observed for terbinafine > butenafine > lanoconazole > griseofulvin, respectively. Terbinafine and griseofulvin had the lowest and highest geometric mean MICs which were 0.01 and 1.23 μg/ml for *T. interdigitale* and *E. floccosum*, respectively.

**TABLE 4:** Susceptibility of 95 clinical isolates and four reference strains of dermatophytes to four antifungal agents.

**Dermatophyte**	**Antifungal drug**	**MIC (μg/ml)**			
		**Range**	**50**	**90**	***G* mean**
*T. interdigitale/T. mentagrophytes* complex (*n* = 27)	Terbinafine	0.003–0.25	0.01	0.03	0.01
	Griseofulvin	0.03–64	0.12	32.2	0.41
	Lanoconazole	0.03–0.5	0.06	0.5	0.09
	Butenafine	0.03–0.5	0.06	0.5	0.08
*T. rubrum* (*n* = 20)	Terbinafine	0.003–1	0.02	1.01	0.03
	Griseofulvin	0.06–64	0.25	51.6	0.54
	Lanoconazole	0.03–1	0.25	1	0.21
	Butenafine	0.03–4	0.12	0.9	0.16
*T. tonsurans* (*n* = 29)	Terbinafine	0.003–0.5	0.003	0.125	0.02
	Griseofulvin	0.03–64	0.25	29.8	0.39
	Lanoconazole	0.03–0.5	0.25	0.5	0.16
	Butenafine	0.01–0.5	0.06	0.5	0.07
*E. floccosum* (*n* = 23)	Terbinafine	0.003–1	0.02	0.168	0.02
	Griseofulvin	0.03–64	1	64	1.23
	Lanoconazole	0.03–0.5	0.25	0.5	0.20
	Butenafine	0.01–0.25	0.03	0.22	0.03

*MIC, minimal inhibitory concentration; G mean, geometric mean.*

## Discussion

Multilocus sequence typing is one of the widely used methods for epidemiological studies and the evolution of microorganisms. Accordingly, in this study, a new MLST scheme was developed based on sequence polymorphisms of seven housekeeping genes *BT2*, *TEF-1*α, *CaM*, *HSP70*, *ACT*, ITS, and D1/D2 of the five major species of dermatophytes. One of the objectives of this study was to cast a light on the genetic diversity of major species of dermatophytes to use MLST. These techniques have shown a low intraspecific genetic diversity among isolates of *T. rubrum*, *T. tonsurans*, and *E. floccosum*. The 64 genotypes obtained from 99 strains identified by MLST (Supplementary Table 2) were grouped in 34 STs. A few alleles were found in all the genes, except for the *HSP70* that had only one genotype. As confirmed by similar studies (Makimura et al., 1999; Persinoti et al., 2018; Zhan et al., 2018), very few genetic changes were observed in the current study. Low levels of polymorphism may be due to the following: (i) the mutation rates in dermatophyte species and (ii) the species recent emergence from a genotype(s).

Species defined by molecular studies do not always correspond to the existing concepts obtained by the ecological and clinical principles. *T. interdigitale* and *T. mentagrophytes* share similar morphological characteristics (macro/microscopic features). In the study of de Hoog et al. (2017), four gene regions were sequenced (β*-tubulin*, ITS, and LSU loci of the rDNA, and the ribosomal 60S protein). Outcomes of the aforementioned study showed a new taxonomy for dermatophytes. *T. interdigitale* only included the anthropophilic species, and zoophilic *T. interdigitale* isolates belong to the species *T. mentagrophytes*. The study of Pchelin et al. (2016) on sequencing four *T. mentagrophytes* genomes showed that *T. mentagrophytes* and *T. interdigitale* belong to the same phylogenetic species. In this study, according to the overall average obtained from intraspecies and interspecies pairwise distances of combination concerning the seven loci (Supplementary Table 3), *T. interdigitale* and *T. mentagrophytes* were regarded as species complexes which may be affected by the epigenetic change during the localization on the animal and human body. Furthermore, they likely share a common ancestor and *T. interdigitale* species are descendants of a *T. mentagrophytes* species. This study confirms the results of the study undertaken by Pchelin et al. (2016).

Whole-genome sequencing of *T. rubrum* and *T. violaceum* showed that a high difference between the two species is found in adhesion genes, which can be the reason for the different localizations on the human body (Zhan et al., 2018). Analysis of sequencing 99 isolates in this study and 22 sequences deposited in the GenBank confirms that these two species belong to the same complex, and *T. violaceum* is derived from *T. rubrum*. Also, the phylogenetic analyses and pairwise distances of the combination of the three loci showed that *T. tonsurans* and *T. equinum* could be regarded as a species complex, where *T. equinum* is derived from *T. tonsurans*.

The phylogenetic tree constructed from the combination of the three genes shows a new topological pattern that confirms the derivation of the anthropophilic and zoophilic genera from the geophilic genus. Besides that, it is possible that *Epidermophyton* was derived from *Nannizzia* and has lost the ability to produce microconidia over time. Analysis of multilocus datasets showed that the name change from Microsporum *racemosum* to *Microsporum cookei* was performed correctly (high support: 100).

Ahmadi et al. (2016) found one *CaM* genotype among *T. tonsurans* and *E. floccosum* isolates and two genotypes among *T. rubrum*, while among isolates of *T. interdigitale*, four genotypes were observed. In the present study, similar to the study of Ahmadi et al. (2016), the most genotype was found in *T. interdigitale* strains. Also, *T. rubrum*, *T. tonsurans*, and *E. floccosum* species did not follow the intraspecies variation. These results can be affected by the number and isolate examined. In a similar vein, the study conducted by Persinoti et al. (2018) confirms the results of this study. The study led by Li et al. (2008) showed that when the D1/D2 and ITS regions were used, most of the species identified wrongly as the *T. mentagrophytes* was reported to be *T. interdigitale*, which supports the findings of the current study.

Another purpose of this study was to evaluate the genes used for the MLST approach. In the present experiment, the housekeeping gene, which has fewer genotypes, is not suitable for MLST, such as the *HSP70*, D1/D2, and *CaM* genes. The study of Zhan et al. (2018) showed that *TUB2* and *RP 60S L1* genes share average performances, while LSU and *TEF3* have a very poor performance. The results of the current research work showed that *BT2, TEF-1*α, and ITS genes are suitable genes for MLST.

In the present study, the highest frequency of ITS genotypes was related to genotypes II and II^∗^ of *T. interdigitale* and *T. mentagrophytes*, respectively. Also, two new genotypes XXVII and XXVIII were introduced for *T. interdigitale* and *T. mentagrophytes*. Another objective of this study was to determine the ST dermatophyte species. The present study was the first to focus on the determination of the ST dermatophyte species. The determination of genotype and ST is useful and important in epidemiological studies of the dermatophyte species. Considering that the relationship between STs and taxa was seen in the combination of three genes (ITS, *BT2*, and *TEF*-*1*α), it seems that the combination of these three genes to determine ST is more appropriate than the combination of seven genes used in this study. Epidemiology studies to improve the basic knowledge of dermatophyte, knowledge into the how of spreading, and understanding the major clones reported from different parts of the world as well as finding the ancestry of isolates can be put into practice. Previous studies on the fungal species gave conflicting reports about the relationship between the genotype and different anatomical sources of the isolates or genotype and the antifungal susceptibility pattern (Odds et al., 2007; Amanloo et al., 2017; Taghipour et al., 2019). A study conducted by Taghipour concerning the determination of the *T. mentagrophytes*/*T. interdigitale* genotype belonging to the rDNA ITS regions showed that there was an association between the geographic locations and clinical presentations (Taghipour et al., 2019). In the present study, such a relationship was not observed due to low genotypes diversity and low number of *T. mentagrophytes* species. In the current study, according to the Fisher’s exact test, no statistically significant association was found between the anatomical source and demographic information with STs and genotypes. Interestingly, genotypic variation was observed for multiple terbinafine-susceptible isolates; therefore, genotypic variation may not always be accompanied by resistance development. Besides, no statistically significant association was found between the antifungal drug susceptibility profile and demographic information with STs and genotypes. However, these associations may have been influenced by confounding variables, such as the number of species, in the determination of STs in epidemiological studies.

The eBURST analysis was performed for all species. Studies of this type are scarce and there is no database for MLST dermatophytes; thus, it was not feasible to compare the genotypes in the current study with those of the other studies. This study was a new starting point to determine the ST and foundation for a dermatophyte MLST database.

A synonymous substitution is a nucleotide mutation which does not alter the amino acid sequence (silent mutation), while in a non-synonymous substitution, a nucleotide mutation manipulates the amino acid sequence of a protein and biological change in the organism. Low ratio of dN/dS indicates that mutations occur at the critical points and these changes lead to a negative or purifying selection. It is believed that the variability among non-coding sequences was higher than that of the coding sequences, but it is striking to note that in the species that were studied, dS occurred more frequently than dN and the ratio of dN/dS was below 1. Considering that, in this study, half of the nucleotide changes led to amino acid changes (16 of 32), it seems that these numbers are justifiable.

According to our results, the MIC of griseofulvin against 99 dermatophyte isolates was in the range of 0.03–64 μg/ml. *T. tonsurans* was the most susceptible species to griseofulvin (MIC_50_ 0.25 μg/ml). GRI showed the reduced susceptibility to *E. floccosum* (MIC_50_, 1 μg/ml). Among 99 tested isolates, three showed a reduced terbinafine susceptibility. Also, 5% of those isolates were cross-resistant to terbinafine and griseofulvin.

The limitations of our study were the retrospective nature of the analysis followed by the lack which is due to underestimation of fungal-related infections in Iran. Overall, the information gathered from seven genes analysis suggests that they were excellent phylogenetic markers for the species level of dermatophytes, although the *CaM* gene was unable to separate the *T. mentagrophytes/T. interdigitale* complex from *T. tonsurans* species in the dendrogram. The most suitable genes for MLST of major pathogenic dermatophytes are the *TEF-1*α, *BT2*, and ITS genes. Also, two new ITS genotypes were found, which had no matches in the GenBank dataset. Besides, data obtained from MLST clarify the status of species: (i) species that are closely related genetically, such as the *T. rubrum*/*T. violaceum* and *T. equinum*/*T. tonsurans* were regarded as a species complex. (ii) It is possible that *Epidermophyton* was derived from *Nannizzia* and then failed to produce microconidia. (iii) The most informative locus was *TEF-1*α.

Considering that there is no database for MLST dermatophytes, it seems necessary to study another gene with a high polymorphism to select the genes used for MLST.

## Data Availability Statement

The original contributions presented in the study are included in the article/Supplementary Material, further inquiries can be directed to the corresponding author/s.

## Author Contributions

MS-G conceived, designed, and supervised the study. ZS performed the experiments and contributed to the analysis of the data. MR-A contributed to the analysis of the data. All authors prepared and wrote the manuscript.

## Conflict of Interest

The authors declare that the research was conducted in the absence of any commercial or financial relationships that could be construed as a potential conflict of interest.

## Footnotes

1 http://eburst.mlst.net/
