# Supplementary material for: Molecular Epidemiology, Genetic Diversity, and Antifungal Susceptibility of Major Pathogenic Dermatophytes Isolated From Human Dermatophytosis
Source: Front Microbiol. 2021 Jun 4;12:643509. doi: 10.3389/fmicb.2021.643509 (PMC8213211; doi:10.3389/fmicb.2021.643509)
Supplement: Supplementary file 1 [file Data_Sheet_1.DOCX]

**Table S1** List of demographic characteristics of dermatophytes isolates in this study

| Species | Code | Isolates no. | Age/  Sex | Source |
| --- | --- | --- | --- | --- |
| *T. interdigitale* | *TI 1* | 93_215 | M/58 | Tinea pedis |
|  | *TI 2* | 93_471 | M/58 | Tinea pedis |
|  | *TI 3* | 91_339 | M/51 | Tinea pedis |
|  | *TI 4* | 89_545 | M/42 | Tinea pedis |
|  | *TI 5* | 91_476 | M/54 | Tinea pedis |
|  | *TI 6* | PTCC 5054 | - | PTCC 5054 |
|  | *TI 7* | 93_214 | M/60 | Tinea pedis |
|  | *TI 8* | 93_379 | M/54 | Tinea pedis |
|  | *TI 9* | 93_408 | M/56 | Tinea pedis |
|  | *TI 10* | 93_890 | M/26 | Tinea pedis |
|  | *TI 11* | 93_274 | M/65 | Tinea pedis |
|  | *TI 12* | 91_485 | M/66 | Tinea pedis |
|  | *TI 13* | 93_417 | M/51 | Tinea pedis |
|  | *TI 14* | 93_742 | M/42 | Tinea pedis |
|  | *TI 15* | 94_205 | M/66 | Tinea cruris |
|  | *TI 16* | 90_987 | M/42 | Tinea pedis |
|  | *TI 17* | 93_653 | M/38 | Tinea pedis |
|  | *TI 18* | 91_363 | M/49 | Tinea pedis |
|  | *TI 19* | 93_2721 | M/47 | Tinea pedis |
|  | *TI 20* | 93_604 | M/40 | Tinea pedis |
|  | *TI 21* | 91_232 | M/30 | Tinea pedis |
| *T. mentagrophytes* | *TM 1* | 92_330 | M/64 | Tinea pedis |
|  | *TM 2* | 89_1098 | M/62 | Tinea pedis |
|  | *TM 3* | 93_472 | M/59 | Tinea pedis |
|  | *TM 4* | 93_1934 | M/30 | Tinea cruris |
|  | *TM 5* | 93_2349 | F/27 | Tinea corporis |
|  | *TM 6* | 93_577 | M/42 | Tinea pedis |
| *T. rubrum* | *TR 1* | 91_386 | F/31 | Tinea cruris |
|  | *TR 2* | 91_384 | F/31 | Tinea corporis |
|  | *TR 3* | 92_2688 | M/45 | Tinea cruris |
|  | *TR 4* | 93_479 | M/28 | Tinea mannum |
|  | *TR 5* | PTCC 5143 | - | PTCC 5143 |
|  | *TR 6* | 93_1842 | M/42 | Tinea cruris |
|  | *TR 7* | 92_398 | M/72 | Tinea cruris |
|  | *TR 8* | 93_1739 | F/38 | Tinea corporis |
|  | *TR 9* | 92_2422 | F/44 | Tinea corporis |
|  | *TR 10* | 90_958 | F/57 | Tinea corporis |
|  | *TR 11* | 93_2414 | M/27 | Tinea corporis |
|  | *TR 12* | 93_478 | M/27 | Tinea cruris |
|  | *TR 13* | 91_704 | F/28 | Tinea cruris |
|  | *TR 14* | 93_1792 | F/31 | Tinea corporis |
|  | *TR 15* | 93_2365 | F/65 | Tinea cruris |
|  | *TR 16* | 90_976 | M/62 | Tinea pedis |
|  | *TR 17* | 89_325 | M/50 | Tinea pedis |
|  | *TR 18* | 94_11 | M/41 | Tinea corporis |
|  | *TR 19* | 89_610 | F/58 | Tinea pedis |
|  | *TR 20* | 93_2824 | M/25 | Tinea cruris |
| *T.tonsurans* | *TT 1* | 93_645 | M/26 | Tinea cruris |
|  | *TT 2* | 93_9 | F/42 | Tinea corporis |
|  | *TT 3* | 93_574 | M/4 | Tinea faciei |
|  | *TT 4* | 93_91 | M/2 | Tinea corporis |
|  | *TT 5* | 93_2543 | M/11 | Tinea capitis |
|  | *TT 6* | 93_2322 | M/17 | Tinea capitis |
|  | *TT 7* | 93_2415 | F/21 | Tinea corporis |
|  | *TT 8* | 93_2392 | M/27 | Tinea pedis |
|  | *TT 9* | 93_2247 | M/10 | Tinea capitis |
|  | *TT 10* | 93_2761 | M/45 | Tinea mannum |
|  | *TT 11* | 93_2407 | M/16 | Tinea faciei |
|  | *TT 12* | 93_486 | M/10 | Tinea capitis |
|  | *TT 13* | 93_412 | M/12 | Tinea capitis |
|  | *TT 14* | 93_457 | M/11 | Tinea capitis |
|  | *TT 15* | CBS 130924 | - | CBS 130924 |
|  | *TT 16* | 92_429 | M/16 | Tinea capitis |
|  | *TT 17* | 93_2892 | M/15 | Tinea corporis |
|  | *TT 18* | 93_2582 | M/7 | Tinea faciei |
|  | *TT 19* | 92_2778 | M/20 | Tinea corporis |
|  | *TT 20* | 93_2809 | M/15 | Tinea corporis |
|  | *TT 21* | 93_430 | F/9 | Tinea capitis |
|  | *TT 22* | 93_306 | M/23 | Tinea corporis |
|  | *TT 23* | 94_80 | M/16 | Tinea corporis |
|  | *TT 24* | 93_2733 | M/26 | Tinea cruris |
|  | *TT 25* | 92_2745 | M/15 | Tinea corporis |
|  | *TT 26* | 93_636 | M/17 | Tinea faciei |
|  | *TT 27* | 92_769 | M/12 | Tinea capitis |
|  | *TT 28* | 89_523 | F/23 | Tinea corporis |
|  | *TT 29* | 93_2345 | F/27 | Tinea faciei |
| *E.floccusum* | *EF 1* | 93-2566 | F/33 | Tinea cruris |
|  | *EF 2* | 93-2070 | M/30 | Tinea corporis |
|  | *EF 3* | 93-2510 | M/48 | Tinea cruris |
|  | *EF 4* | CBS 767.73 | - | CBS 767.73 |
|  | *EF 5* | 93-2682 | M/31 | Tinea cruris |
|  | *EF 6* | 93-2286 | F/65 | Tinea cruris |
|  | *EF 7* | 93-2432 | M/23 | Tinea cruris |
|  | *EF 8* | 93-2767 | F/40 | Tinea corporis |
|  | *EF 9* | 93-2534 | M/47 | Tinea cruris |
|  | *EF 10* | 93-2645 | F/46 | Tinea corporis |
|  | *EF 11* | 89-680 | M/52 | Tinea pedis |
|  | *EF 12* | 93-1889 | M/30 | Tinea cruris |
|  | *EF 13* | 93-1717 | M/25 | Tinea cruris |
|  | *EF 14* | 93-2068 | M/30 | Tinea cruris |
|  | *EF 15* | 93-1952 | F/39 | Tinea corporis |
|  | *EF 16* | 93-2689 | M/33 | Tinea cruris |
|  | *EF 17* | 93-1642 | M/25 | Tinea cruris |
|  | *EF 18* | 93-1787 | M/26 | Tinea cruris |
|  | *EF 19* | 94-483 | M/23 | Tinea cruris |
|  | *EF 20* | 93-1757 | M/49 | Tinea cruris |
|  | *EF 21* | 93-1856 | M/45 | Tinea cruris |
|  | *EF 22* | 93-2087 | F/47 | Tinea cruris |
|  | *EF 23* | 93-983 | F/77 | Tinea corporis |
| M, Male; F, Female; | | | | |
