# Supplementary material for: Molecular Epidemiology, Genetic Diversity, and Antifungal Susceptibility of Major Pathogenic Dermatophytes Isolated From Human Dermatophytosis
Source: Front Microbiol. 2021 Jun 4;12:643509. doi: 10.3389/fmicb.2021.643509 (PMC8213211; doi:10.3389/fmicb.2021.643509)
Supplement: Supplementary file 2 [file Data_Sheet_2.DOC]

**Table S2** List of dermatophytes isolates showing GenBank accession no. and the genotype of seven loci and sequence types

| **Species** | **Isolates no.** | **ITS** | ***BT2*** | ***TEF-1α*** | ***ACT*** | ***CaM*** | ***HSP70*** | **D1/D2** | **ST (allelic profile, 7 genes)** | **ST (allelic**  **profile, 3 genes)** |
| --- | --- | --- | --- | --- | --- | --- | --- | --- | --- | --- |
| ***T. interdigitale*** | 93_215 | MF109062 | MG251820 | - | MH523163 | MH523252 | - | - | 1 ( II ,1,1,2,2,1,3) | 1 ( II ,1,1) |
|  | 93_471 | MF109042 | MG251801 | MG099972 | MH523159 | MH523248 | - | MH389748 | 1 ( II ,1,1,2,2,1,3) | 1 ( II ,1,1) |
|  | 91_339 | MF109049 | MG251808 | - | MH523157 | MH523244 | MH523335 | MH389744 | 1 ( II ,1,1,2,2,1,3) | 1 ( II ,1,1) |
|  | 89_545 | MF109060 | - | MG099968 | - | MH523256 | - | - | 1 ( II ,1,1,2,2,1,3) | 1 ( II ,1,1) |
|  | 91_476 | MF109059 | MG251818 | MG099967 | MH523166 | MH523255 | - | - | 1 ( II ,1,1,2,2,1,3) | 1 ( II ,1,1) |
|  | PTCC 5054 | MF109050 | - | - | MH523156 | MH523243 | - | MH389743 | 1 ( II ,1,1,2,2,1,3) | 1 ( II ,1,1) |
|  | 93_214 | MF109037 | MG251798 | MG099969 | - | - | - | - | 2 ( XXVII,1,1,2,2,1,2) | 2 (XXVII,1,1) |
|  | 93_379 | MF109041 | MG251800 | MG099971 | MH523160 | MH523249 | - | MH389749 | 3 ( II ,1,1,2,2,1,1) | 1 ( II ,1,1) |
|  | 93_408 | MF109053 | MG251812 | - | MH523164 | MH523253 | - | - | 3 ( II ,1,1,2,2,1,1) | 1 ( II ,1,1) |
|  | 93_890 | MF109054 | MG251813 | - | - | - | - | - | 3 ( II ,1,1,2,2,1,1) | 1 ( II ,1,1) |
|  | 93_274 | MF109038 | MG251799 | - | MH523162 | MH523251 | - | - | 3 ( II,1,1,2,2,1,1) | 1 ( II,1,1) |
|  | 91_485 | MF109056 | MG251815 | - | MH523165 | MH523254 | - | - | 3 ( II,1,1,2,2,1,1) | 1 ( II,1,1) |
|  | 93_417 | MF109039 | - | MG099970 | - | - | - | - | 4 (XXVII,2,1,2,2,1,2) | 3 (XXVII,2,1) |
|  | 93_742 | MF109043 | MG251802 | - | - | MH523247 | - | MH389747 | 5 ( II,1,1,1,2,1,1) | 1 ( II,1,1) |
|  | 94_205 | MF109061 | MG251819 | - | MH523153 | - | H523333 | MH389740 | 6 ( II,1,4,2,1,1,1) | 4 ( II,1,4) |
|  | 90_987 | MF109040 | - | - | MH523161 | MH523250 | - | MH389750 | 7 ( II,3,1,2,2,1,3) | 5 ( II,3,1) |
|  | 93_653 | MF109047 | MG251806 | - | - | MH523246 | MH523337 | MH389746 | 8 ( II,1,3,2,2,1,1) | 6 ( II,1,3) |
|  | 91_363 | MF109048 | MG251807 | - | MH523158 | MH523245 | MH523336 | MH389745 | 9 ( II,1,4,2,2,1,1) | 4 ( II,1,4) |
|  | 3_2721 | MF109051 | MG251810 | - | MH523155 | MH523242 | MH523334 | MH389742 | 10 ( II,1,2,2,2,1,3) | 7 ( II,1,2) |
|  | 93_604 | MF109052 | MG251811 | - | MH523154 | MH523241 | - | MH389741 | 11 ( II,1,5,2,2,1,1) | 8 ( II,1,5) |
|  | 91_232 | MF109055 | MG251814 | MG099964 | MH523152 | - | H523332 | MH389739 | 12 (II,1,1,2,1,1,1) | 1 (II,1,1) |
| ***T. mentagrophytes*** | 92_330 | MH356569 | - | MW592745 | MH745938 | MH745951 | H745951 | - | 1 (II*,1,2,1,1,1,2) | 1 (II*,1,2) |
|  | 9_1098 | MH356566 | - | - | MH745935 | MH745955 | H745948 | - | 2 ( II*,1,2,1,1,1,1) | 1 ( II*,1,2) |
|  | 93_472 | MH356570 | MW592747 | - | - | MH745954 | - | - | 3(XXVIII,1,2,1,1,1,1) | 2(XXVIII,1,2) |
|  | 93_1934 | MH356567 | - | MW592746 | MH745937 | MH745952 | H745947 | - | 4 ( II*,1,1,1,1,1,1) | 3 ( II*,1,1) |
|  | 93_2349 | MH356568 | - | - | MH745936 | MH745953 | - | - | 5 ( II*,1,2,1,1,1,2) | 1 ( II*,1,2) |
|  | 93_577 | MH356571 | MW592748 | - | MH745939 | MH745950 | MH745949 | MH745111 | 5 ( II*,1,2,1,1,1,2) | 1 ( II*,1,2) |
| ***T. rubrum*** | 91_386 | MF155596 | MG251832 | MG251752 | MH523180 | MH523269 | - | MH392579 | 1 (1,1,1,1,1,1,1) | 1 (1,1,1) |
|  | 91_384 | MF155591 | MG251829 | MG251753 | MH523173 | MH523262 | MH523340 | MH392572 | 1 (1,1,1,1,1,1,1) | 1 (1,1,1) |
|  | 2_2688 | MF155592 | MG251830 | - | - | MH523277 | - | - | 1 (1,1,1,1,1,1,1) | 1 (1,1,1) |
|  | 93_479 | MF155593 | - | MG251754 | MH523172 | MH523260 | MH523338 | MH392570 | 1 (1,1,1,1,1,1,1) | 1 (1,1,1) |
|  | PTCC 5143 | MF155590 | MG251828 | - | - | - | - | - | 1 (1,1,1,1,1,1,1) | 1 (1,1,1) |
|  | 3_1842 | MF155595 | MG251831 | MG251757 | - | - | - | - | 1 (1,1,1,1,1,1,1) | 1 (1,1,1) |
|  | 92_398 | MF155577 | - | MG251743 | MH523182 | MH523271 | - | MH392581 | 1 (1,1,1,1,1,1,1) | 1 (1,1,1) |
|  | 3_1739 | MF155578 | MG251821 | MG251744 | MH523183 | MH523271 | - | MH392582 | 1 (1,1,1,1,1,1,1) | 1 (1,1,1) |
|  | 2_2422 | MF155579 | MG251822 | MG251745 | MH523181 | MH523270 | - | MH392580 | 1 (1,1,1,1,1,1,1) | 1 (1,1,1) |
|  | 90_958 | MF155580 | MG251823 | MG251746 | MH523184 | - | - | - | 1 (1,1,1,1,1,1,1) | 1 (1,1,1) |
|  | 3_2414 | MF155581 | MG251824 | MG251747 | MH523179 | MH523268 | - | MH392578 | 1 (1,1,1,1,1,1,1) | 1 (1,1,1) |
|  | 93_478 | MF155582 | - | MG251748 | MH523179 | MH523267 | - | MH392577 | 1 (1,1,1,1,1,1,1) | 1 (1,1,1) |
|  | 91_704 | MF155583 | MG251825 | MG251749 | MH523177 | MH523266 | - | MH392576 | 1 (1,1,1,1,1,1,1) | 1 (1,1,1) |
|  | 3_1792 | MF155584 | MG251826 | MG251756 | - | MH523279 | MH523343 | - | 1 (1,1,1,1,1,1,1) | 1 (1,1,1) |
|  | 3_2365 | - | - | - | - | - | - | - | 1 (1,1,1,1,1,1,1) | 1 (1,1,1) |
|  | 90_976 | MF155594 | - | MG251755 | - | MH523261 | H523339 | MH392571 | 2 (1,1,1,2,1,1,1) | 1 (1,1,1) |
|  | 89_325 | MF155586 | - | MG251751 | MH523175 | MH523264 | H523342 | MH392574 | 3 (1,2,1,1,1,1,1) | 2 (1,2,1) |
|  | 94_11 | MF155587 | - | - | MH523174 | MH523263 | H523341 | MH392573 | 4 (1,3,3,1,1,1,1) | 3 (1,3,3) |
|  | 89_610 | MF155589 | - | - | - | - | - | - | 5 (1,1,2,1,1,1,1) | 4 (1,1,2) |
|  | 3_2824 | MF155588 | MG251827 | - | - | MH523278 | - | - | 6 (1,1,3,1,1,1,1) | 5 (1,1,3) |
| ***T. tonsurans*** | 93_645 | MF158259 | MG267094 | MG251770 | - | MH523304 | - | MH392615 | 1 (1,1,1,1,1,1,1) | 1 (1,1,1) |
|  | 93_9 | MF158260 | MG267095 | MG251765 | - | MH523305 | - | MH392616 | 1 (1,1,1,1,1,1,1) | 1 (1,1,1) |
|  | 93_574 | MF158261 | MG267096 | MG251764 | - | MH523303 | - | MH392614 | 1 (1,1,1,1,1,1,1) | 1 (1,1,1) |
|  | 93_91 | MF158262 | MG267097 | MG251763 | - | MH523302 | - | MH392613 | 1 (1,1,1,1,1,1,1) | 1 (1,1,1) |
|  | 3_2543 | MF158263 | MG267098 | MG251762 | - | MH523301 | - | - | 1 (1,1,1,1,1,1,1) | 1 (1,1,1) |
|  | 3_2322 | MF158264 | MG267099 | MG251761 | - | MH523300 | - | - | 1 (1,1,1,1,1,1,1) | 1 (1,1,1) |
|  | 3_2415 | - | - | - | - | - | - | MH392610 | 1 (1,1,1,1,1,1,1) | 1 (1,1,1) |
|  | 3_2392 | MF158266 | MG267100 | MG251772 | - | - | - | MH392609 | 1 (1,1,1,1,1,1,1) | 1 (1,1,1) |
|  | 3_2247 | MF158267 | MG267101 | MG251759 | - | - | - | - | 1 (1,1,1,1,1,1,1) | 1 (1,1,1) |
|  | 3_2761 | MF158269 | MG267103 | MG251778 | - | - | - | - | 1 (1,1,1,1,1,1,1) | 1 (1,1,1) |
|  | 3_2407 | MF158270 | MG267104 | MG251777 | - | - | - | - | 1 (1,1,1,1,1,1,1) | 1 (1,1,1) |
|  | 93_486 | MF158271 | MG267105 | MG251776 | - | - | - | - | 1 (1,1,1,1,1,1,1) | 1 (1,1,1) |
|  | 93_412 | MF158272 | MG267106 | MG251775 | - | - | - | - | 1 (1,1,1,1,1,1,1) | 1 (1,1,1) |
|  | 93_457 | MF158273 | MG267107 | MG251774 | - | MH523291 | - | - | 1 (1,1,1,1,1,1,1) | 1 (1,1,1) |
|  | CBS 130924 | MW584278 | - | - | - | - | - | - | 1 (1,1,1,1,1,1,1) | 1 (1,1,1) |
|  | 92_429 | MF158276 | MG267109 | - | MH523200 | MH523288 | - | MH392599 | 1 (1,1,1,1,1,1,1) | 1 (1,1,1) |
|  | 3_2892 | MF158277 | MG267110 | - | MH523191 | MH523308 | - | MH392598 | 1 (1,1,1,1,1,1,1) | 1 (1,1,1) |
|  | 3_2582 | MF158278 | MG267111 | - | MH523198 | MH523287 | - | MH392597 | 1 (1,1,1,1,1,1,1) | 1 (1,1,1) |
|  | 2_2778 | MF158280 | MG267113 | MG251769 | MH523196 | MH523285 | MH523349 | MH392595 | 1 (1,1,1,1,1,1,1) | 1 (1,1,1) |
|  | 3_2809 | MF158281 | MG267114 | MG251768 | - | MH523307 | - | MH392618 | 1 (1,1,1,1,1,1,1) | 1 (1,1,1) |
|  | 93_430 | MF158282 | MG267115 | - | MH523195 | MH523284 | MH523348 | MH392594 | 1 (1,1,1,1,1,1,1) | 1 (1,1,1) |
|  | 93_306 | MF158284 | MG267116 | MG251766 | MH523193 | MH523282 | MH523346 | MH392592 | 1 (1,1,1,1,1,1,1) | 1 (1,1,1) |
|  | 94_80 | MF158286 | MG267117 | - | MH523191 | MH523280 | MH523344 | MH392590 | 1 (1,1,1,1,1,1,1) | 1 (1,1,1) |
|  | 3_2733 | MF158274 | MG267119 | - | MH523202 | MH523290 | - | - | 1 (1,1,1,1,1,1,1) | 1 (1,1,1) |
|  | 2_2745 | MF158279 | MG267112 | - | MH523197 | MH523286 | - | MH392596 | 2 (1,1,1,1,1,1,1) | 1 (1,1,1) |
|  | 93_636 | MF158283 | - | MG251767 | MH523194 | MH523283 | MH523347 | MH392593 | 3 (1,2,1,1,1,1,1) | 2 (1,2,1) |
|  | 92_769 | MF158285 | MG267118 | - | - | MH523306 | - | MH392617 | 4 (1,1,4,1,1,1,1) | 3 (1,1,4) |
|  | 89_523 | MF158287 | - | - | MH523192 | MH523281 | H523345 | MH392591 | 5 (1,1,2,1,1,1,1) | 4 (1,1,2) |
|  | 3_2345 | MF158268 | MG267102 | - | - | - | - | - | 6 (1,1,3,1,1,1,1) | 5 (1,1,3) |
| ***E. floccusum*** | 93-2566 | MF158289 | - | MG251795 | - | - | - | MH389737 | 1 (1,1,1,1,1,1,1) | 1 (1,1,1) |
|  | 93-2070 |  | MG267093 | MG251779 | - | - | - | MH389738 | 1 (1,1,1,1,1,1,1) | 1 (1,1,1) |
|  | 93-2510 | MF158304 | MG267087 | - | MH523225 | MH523314 | - | - | 2 (1,1,3,2,1,1,1) | 2 (1,1,3) |
|  | CBS 767.73 | - | - | - | - | - | - | - | 3 (1,1,1,2,1,1,1) | 1 (1,1,1) |
|  | 93-2682 | MF158306 | MG267089 | MG251783 | MH523223 | MH523312 | H523353 | MH389719 | 3 (1,1,1,2,1,1,1) | 1 (1,1,1) |
|  | 93-2286 | MF158307 | MG267090 | MG251782 | MH523222 | MH523311 | H523352 | MH389718 | 3 (1,1,1,2,1,1,1) | 1 (1,1,1) |
|  | 93-2432 | MF158308 | MG267091 | MG251781 | MH523220 | MH523309 | H523350 | MH389716 | 3 (1,1,1,2,1,1,1) | 1 (1,1,1) |
|  | 93-2767 | MF158309 | MG267092 | MG251780 | MH523221 | MH523310 | MH523351 | MH389717 | 3 (1,1,1,2,1,1,1) | 1 (1,1,1) |
|  | 93-2534 | MF158303 | - | MG251785 | MH523226 | MH523315 | - | MH389722 | 3 (1,1,1,2,1,1,1) | 1 (1,1,1) |
|  | 93-2645 | MF158288 | - | MG251796 | - |  | - | MH389736 | 3 (1,1,1,2,1,1,1) | 1 (1,1,1) |
|  | 89-680 | MF158300 | MG267086 | - | MH523228 | MH523317 | - | MH389724 | 3 (1,1,1,2,1,1,1) | 1 (1,1,1) |
|  | 93-1889 | MF158290 | MG267077 | MG251794 | - | - | - | - | 3 (1,1,1,2,1,1,1) | 1 (1,1,1) |
|  | 93-1717 | - | - | - | - | - | - | - | 3 (1,1,1,2,1,1,1) | 1 (1,1,1) |
|  | 93-2068 | MF158292 | MG267078 | MG251793 | - | - | - | MH389733 | 3 (1,1,1,2,1,1,1) | 1 (1,1,1) |
|  | 93-1952 | MF158294 | MG267079 | MG251792 | - | MH523323 | - | MH389730 | 3 (1,1,1,2,1,1,1) | 1 (1,1,1) |
|  | 93-2689 | MF158295 | MG267080 | MG251791 | MH523233 | MH523322 | - | - | 3 (1,1,1,2,1,1,1) | 1 (1,1,1) |
|  | 93-1642 | MF158296 | MG267081 | MG251790 | MH523232 | MH523321 | - | - | 3 (1,1,1,2,1,1,1) | 1 (1,1,1) |
|  | 93-1787 | MF158297 | MG267082 | MG251789 | MH523231 | MH523320 | - | - | 3 (1,1,1,2,1,1,1) | 1 (1,1,1) |
|  | 94-483 | MF158301 | MG267085 | - | MH523227 | MH523316 | - | MH389723 | 3 (1,1,1,2,1,1,1) | 1 (1,1,1) |
|  | 93-1757 | MF158298 | MG267083 | MG251788 | MH523230 | MH523319 | - | MH389726 | 3 (1,1,1,2,1,1,1) | 1 (1,1,1) |
|  | 93-1856 | MF158299 | MG267084 | MG251787 | MH523229 | MH523318 | - | MH389725 | 3 (1,1,1,2,1,1,1) | 1 (1,1,1) |
|  | 93-2087 | MF158293 | - | - | - | - | - | MH389732 | 4 (1,1,2,2,1,1,1) | 3 (1,1,2) |
|  | 93-983 | MF158302 | - | MG251786 | - | - | - | MH389731 | 5 (1,2,1,2,1,1,1) | 4 (1,2,1) |
| Allelic profile 7 genes, ITS, *BT2*, *TEF-1α*, *ACT*, *CaM*, *HSP70*, D1/D2; allelic profile 3 genes, ITS, *BT2*, *TEF-1α*. | | | | | | | | | | |
