# Supplementary material for: Molecular Epidemiology, Genetic Diversity, and Antifungal Susceptibility of Major Pathogenic Dermatophytes Isolated From Human Dermatophytosis
Source: Front Microbiol. 2021 Jun 4;12:643509. doi: 10.3389/fmicb.2021.643509 (PMC8213211; doi:10.3389/fmicb.2021.643509)
Supplement: Supplementary file 3 [file Data_Sheet_3.DOC]

| Species | *T. mentagrophytes* | *T. interdigitale* | *T. tonsurans* | *T. rubrum* |
| --- | --- | --- | --- | --- |
| *T. mentagrophytes* | 0.0001 |  |  |  |
| *T. interdigitale* | 0.001 | 0.0001 |  |  |
| *T. tonsurans* | 0.003 | 0.004 | 0.0001 |  |
| *T. rubrum* | 0.028 | 0.028 | 0.027 | 0.0001 |

**Table S3** Pairwise distances count matrix of intra-species and interspecies based on pairwise sequence comparison of combination the seven loci between *Trichophyton* species investigated in this study
